# A new risk score model to predict the presence of significant coronary artery disease in renal transplant candidates

**DOI:** 10.1186/2047-1440-2-18

**Published:** 2013-11-01

**Authors:** Luís Henrique Wolff Gowdak, Flávio Jota de Paula, Luiz Antônio Machado César, Luiz Aparecido Bortolotto, José Jayme Galvão de Lima

**Affiliations:** 1 Heart Institute (InCor), Hospital das Clínicas, University of São Paulo Medical School, Avenida Doutor Enéas de Carvalho Aguiar, 44, São Paulo 05403-000, Brazil; 2 Renal Transplant Unit, Hospital das Clínicas, University of São Paulo Medical School, Avenida Doutor Enéas de Carvalho Aguiar, 255, São Paulo 05403-000, Brazil

**Keywords:** Coronary artery disease, End-stage renal disease, Kidney transplant, Angiography, Risk score

## Abstract

**Background:**

Renal transplant candidates are at high risk of coronary artery disease (CAD). We sought to develop a new risk score model to determine the pre-test probability of the occurrence of significant CAD in renal transplant candidates.

**Methods:**

A total of 1,060 renal transplant candidates underwent a comprehensive cardiovascular risk evaluation. Patients considered at high risk of CAD (age ≥50 years, with either diabetes mellitus (DM) or cardiovascular disease (CVD)), or having noninvasive testing suggestive of CAD were referred for coronary angiography (n = 524). Significant CAD was defined by the presence of luminal stenosis ≥70%. A binary logistic regression model was built, and the resulting logistic regression coefficient B for each variable was multiplied by 10 and rounded to the next whole number. For each patient, a corresponding risk score was calculated and the receiver operating characteristic (ROC) curve was constructed.

**Results:**

The final equation for the model was risk score = (age × 0.4) + (DM × 9) + (CVD × 14) and for the probability of CAD (%) = (risk score × 2) – 23. The corresponding ROC for the accuracy of the diagnosis of CAD was 0.75 (*P* <0.0001) in the developmental model.

**Conclusions:**

We developed a simple clinical risk score to determine the pre-test probability of significant CAD in renal transplant candidates. This model may help those directly involved in the care of patients with end-stage renal disease being considered for transplantation in an attempt to reduce the rate of cardiovascular events that presently hampers the long-term prognosis of such patients.

## Background

It is now widely recognized that patients with any degree of renal dysfunction are at high risk of cardiovascular events [1], particularly due to coronary artery disease (CAD) [2]. Therefore, for patients with advanced chronic kidney disease (CKD) (stage V) who are considered as candidates for renal transplantation, screening for CAD before their inclusion on waiting lists has become a major challenge [3]. The importance of an unmissed diagnosis of CAD in this population is further extended even after a successful, uneventful renal transplant, because according to the United States Renal Data System (USRDS) [4], cardiovascular disease (CVD) persists as the most common cause of death (30%) in renal transplant recipients, again due to the high prevalence of CAD.

The task of correctly ascertaining the presence of CAD can be more overwhelming when we take into account that there is a high prevalence of asymptomatic patients with extensive CAD [5], that the noninvasive testing of CAD in patients with CKD has a reported lower sensitivity/specificity than that of the general population [6], and routine invasive diagnostic coronary angiography is a risky [7] and costly procedure.

Thus, two major transplant associations, the American Society of Transplantation (AST) [8] and the European Renal Association - European Dialysis and Transplant Association (ERA-EDTA) [9] have established their own guidelines for cardiovascular risk assessment among potential kidney transplant recipients. Although they are slightly different, both guidelines have identified three clinical characteristics associated with an intermediate- to high-risk probability of CAD: age ≥50 years, diabetes mellitus (DM), and/or evidence of CVD (symptomatic CAD, previous myocardial infarction, and/or congestive heart failure). The discrimination between low- and high-risk patients for CAD seems straightforward when no clinical risk factors are present; in this scenario, low-risk patients can safely undergo transplantation without further cardiac evaluation [10]. According to these guidelines, patients at intermediate-risk (by ERA-EDTA) or high-risk (by AST) for CAD would undergo noninvasive testing, and if the test yields a positive result, then coronary angiography is warranted. Even though these guidelines have been in use for a decade or so, their impact on the diagnosis of pre-transplant CAD has not been as strong as first assumed; in fact, there is still an open discussion on what should be the best approach to diagnose CAD in high-risk renal transplant candidates [3].

The purpose of the present study was to develop and validate a simple, new risk score model to determine the pre-test probability of any renal transplant candidate of having significant CAD during cardiovascular risk assessment before a patient’s inclusion on waiting lists, based on the presence of the aforementioned risk factors for CAD.

## Patients and methods

### Patient selection and study protocol

Between 1998 and 2012, 1,060 patients with chronic renal disease stage V on maintenance hemodialysis were referred for cardiovascular risk assessment before inclusion on kidney transplant waiting lists. All patients underwent a comprehensive cardiovascular risk evaluation as described elsewhere [2]. Briefly, we obtained from all patients a medical history and performed a physical examination with special interest in evidence of previous and/or current CVD. We also performed resting 12-lead electrocardiography (ECG), transthoracic echocardiography (TTE), and myocardial perfusion scanning by single-photon emission computed tomography (SPECT) Tc^99m^ sestamibi after pharmacological stress with dipyridamole. Patients considered at high risk for CAD (age ≥50 years, or diabetes (types 1 or 2), or having CVD, such as angina, previous myocardial infarction or stroke, left ventricular dysfunction, or extracardiac atherosclerosis), or having noninvasive testing suggestive of CAD (39.5% of the non-diabetic, or without CVD), were eligible for the study. For the purpose of this study, the definition of CVD was widened to include other clinical presentations of atherosclerosis, such as previous stroke or transient ischemic attacks, peripheral artery disease (PAD), or both together. PAD was defined by finding during physical examination an absence of the peripheral distal pulses. Intermittent claudication was not considered for the diagnosis of PAD.

Significant CAD was arbitrarily defined as luminal stenosis ≥70% in one or more epicardial arteries by visual estimation from two independent experts.

Following the study protocol, 524 patients fulfilled the high-risk criteria for CAD and were further referred for coronary angiography, therefore, comprising the study population for the present investigation.

This study was approved by the institutional ethics committee and conducted according to the Declaration of Helsinki [11]. All subjects provided a signed, written informed consent at entry into the study, and all patients agreed with the invasive assessment using coronary angiography. After inception, patients were treated according to the current guidelines for treatment of patients with, or at high-risk for, CVD [12,13] including 72 patients referred for myocardial revascularization procedures (coronary artery bypass grafting (CABG) or percutaneous coronary intervention (PCI)). After cardiovascular risk stratification, patients were referred back to the renal transplant unit for follow-up and inclusion/exclusion in transplant waiting lists.

### Model development

To develop the risk score model, approximately 50% of the study population was randomly selected, whereas the remaining half of the population was used to validate the model. Table 1 shows the main characteristics of patients in the ‘model group’ (n = 259; 49.4%) and in the ‘validation group’ (n = 265; 50.6%).

**Table 1:** Main characteristics of the study population

**Variable**	**Model group (n = 259)**	**Validation group (n = 265)**	***P*** **value**
Age (years)	56.8 ± 8.4	55.8 ± 9.1	0.21
Male gender (%)	69.8	67.3	0.57
Caucasian (%)	65.5	69.5	0.34
Hypertension (%)	89.8	91.1	0.66
Diabetes (%)	46.7	45.7	0.86
Smoking (%)	22.4	23.0	0.92
Overweight/obesity (%)	59.2	52.8	0.14
Dyslipidemia (%)	45.9	39.0	0.13
Previous MI (%)	12.9	11.1	0.59
Angina (%)	25.5	24.2	0.76
Previous stroke (%)	12.5	11.9	0.89
PAD (%)	28.6	29.0	1.00
Heart failure (%)	10.2	13.4	0.28
Significant CAD (%)	45.1	52.4	0.10
Any CVD (%)	45.5	48.7	0.48
BMI (kg/m^2^)	26.1 ± 4.5	25.7 ± 4.7	0.29
SAP (mmHg)	167 ± 31	167 ± 33	0.95
DAP (mmHg)	97 ± 16	96 ± 17	0.41
Glucose level (mg%)	122 ± 64	130 ± 98	0.30
Hematocrit (mg%)	37 ± 5	36 ± 6	0.06
Cholesterol (mg%)	182 ± 47	178 ± 48	0.32
Triglycerides (mg%)	158 ± 100	161 ± 130	0.73
Creatinine (mg%)	8.7 ± 2.7	8.5 ± 2.9	0.40
Time on dialysis (months)	36 ± 37	37 ± 43	0.79
LVEF (%)	62 ± 13	61 ± 12	0.49

BMI, body mass index; CAD, coronary artery disease; CVD, cardiovascular disease; DAP, diastolic arterial pressure; LVEF, left ventricular ejection fraction; MI, myocardial infarction; PAD, peripheral artery disease; SAP, systolic arterial pressure.

A binary logistic regression model was built from the set of three clinically relevant candidate variables, namely age, diabetes (yes/no), and overt CVD (known atherosclerotic disease including CAD, cerebrovascular disease or PAD, or heart failure). Findings of myocardial perfusion scan were not considered in this model since it would defeat the purpose of having a simple, fast, applicable, clinically-based model, without any further testing. The logistic regression coefficient B for each variable was multiplied by 10 and rounded to the next whole number. For each patient, a corresponding risk score was calculated based on the logistic regression model and the area under the receiver operating characteristic (ROC) curve (AUC_ROC_) was constructed for the diagnosis of significant CAD. Finally, the prevalence of significant CAD for each risk score was determined, and a final linear regression model between risk score and the probability of CAD was calculated. All statistical analyses were performed using the commercially available statistical package SPSS, version 13 (SPSS Inc, Chicago, IL, USA).

### Model validation

After the model was completed, the risk score for the remaining patients was calculated from the final model equation based on their individual values for the risk factors. Using this score, sensitivity and false positive fractions were calculated for all possible threshold values, regarding the presence of significant CAD. The AUC_ROC_ was calculated with 95% confidence intervals.

## Results

### The risk score model

Table 2 shows the results of the logistic regression used to develop the new risk score for significant CAD in renal transplant candidates. All three variables entered the model with *P* value <0.05. The corresponding B coefficient was used to assign a specific value as a risk score.

**Table 2:** Variables in the equation

**Variable**	**B**	**SE**	**Wald**	**df**	**Sig**	**Exp(B)**	**Score**
Age	0.039	0.016	5.827	1	0.016	1.040	0.4
DM (+)	0.853	0.278	9.423	1	0.002	2.347	9
CVD (+)	1.392	0.278	25.153	1	0.0001	4.023	14

CVD, cardiovascular disease; df, degrees of freedom; DM, diabetes mellitus; Exp(B), exponentiation of the B coefficient; SE, standard error; Sig, significance.

The final equation for the developmental model is:

(1) $$\begin{array}{l} \mathrm{Risk}\;\mathrm{score}=\left(\mathrm{Age}\times 0.4\right)+\left(\mathrm{DM}\times 9\right)\\ \;+\left(\mathrm{CVD}\times 14\right) \end{array}$$

where age in years, DM/CVD if yes = 1

**Figure 1:**
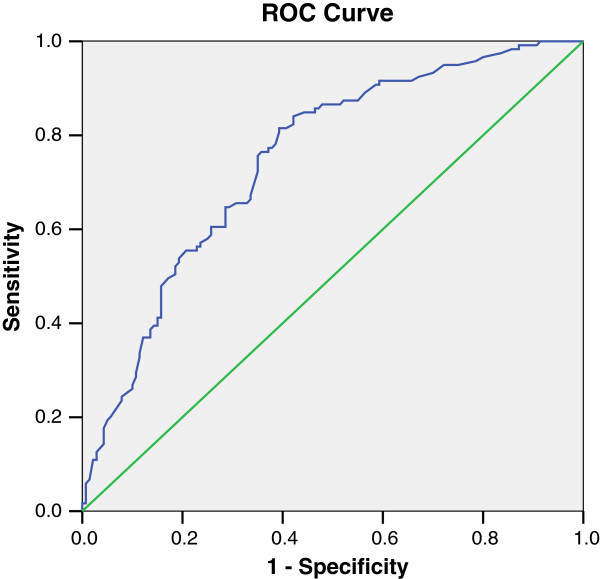
**ROC curve for the new risk score for predicting significant CAD in renal transplant candidates.** CAD, coronary artery disease; ROC, receiver operating characteristic.

Figure 1 shows the corresponding ROC curve and Table 3 shows the AUC_ROC_ for this model.

**Table 3:** **AUC**_**ROC**_ (**developmental model**)

**Area**	**SE(a)**	**Asymptotic sig(b)**	**Asymptotic 95% confidence interval**	
			**Lower bound**	**Upper bound**
0.748	0.030	0.0001	0.689	0.807

AUC_ROC_, area under the receiver operating characteristic curve; SE, standard error; sig, significance.

### The validation model

**Figure 2:**
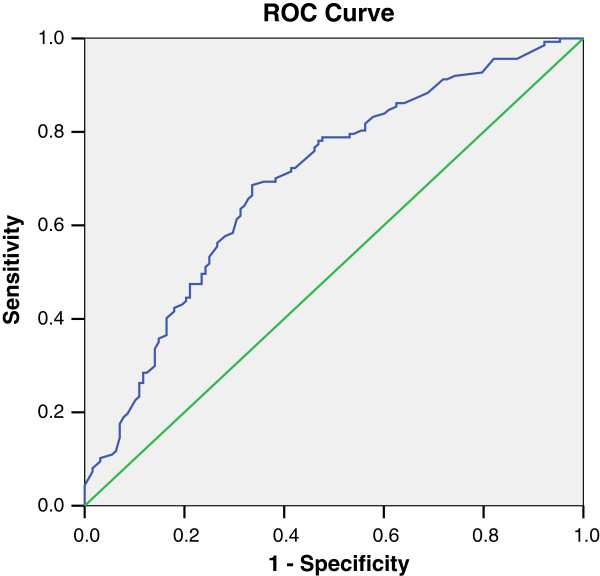
**ROC curve for the new risk score for predicting significant CAD in a different set of renal transplant candidates (validation population).** CAD, coronary artery disease; ROC, receiver operating characteristic.

After establishing the new risk score model, we validated it in a new set of renal transplant candidates. Figure 2 and Table 4 show the results of the new risk score in this population.

**Table 4:** **AUC**_**ROC**_ (**validation model**)

**Area**	**SE(a)**	**Asymptotic sig(b)**	**Asymptotic 95% confidence interval**	
			**Lower bound**	**Upper bound**
0.696	0.032	0.0001	0.633	0.759

AUC_ROC_, area under the receiver operating characteristic curve; SE, standard error; sig, significance.

### Prevalence of significant CAD according to the risk score

**Figure 3:**
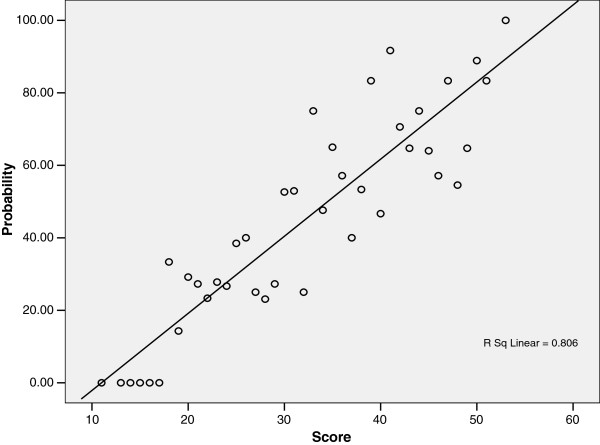
**Scatter plot of the probability of having significant CAD according to risk score.** CAD, coronary artery disease.

To have a final model to predict the probability of significant CAD based only on three clinical variables, we determined the prevalence of significant CAD as disclosed by invasive angiography for each score among renal transplant candidates. Figure 3 shows the resulting scatter plot for patients with different score points and the corresponding prevalence of significant CAD. Note that there is a good relationship between the score and the probability of CAD with a regression coefficient (*R*^2^) of 0.81 (*P* <0.0001).

The resulting equation for the linear model is:

(2) $$\mathrm{Probability}\;\mathrm{of}\;\mathrm{CAD}=\left(\mathrm{Risk}\;-\;\mathrm{score}\times 2\right)-23$$

### The final model

We can combine equations (1) and (2) into a single equation to get the expected probability of any patient with chronic renal disease stage V to have significant CAD during cardiovascular risk assessment before renal transplantation, as follows:

(3) $$\begin{array}{l} \mathrm{Probability}\;\mathrm{of}\;\mathrm{CAD}=\{[\left(\mathrm{Age}\times 0.4\right)+\left(\mathrm{DM}\times 9\right)\\ \;+\;\left(\mathrm{CVD}\times 14\right)]\times 2\}\;-\;23 \end{array}$$

For example, a nondiabetic 40-year-old patient with no evidence of CVD will have an expected probability of having significant CAD of:

$$\begin{array}{c} \mathrm{Probability}\;\mathrm{of}\;\mathrm{CAD}=\left\{\left[\left(40\times 0.4\right)+\left(0\times 9\right)+\left(0\times 14\right)\right]\times 2\right\}\;-\;23\\ \mathrm{Probability}\;\mathrm{of}\;\mathrm{CAD}=9\%\left(6.4-11.6\%\right) \end{array}$$

On the other hand, a 65-year-old diabetic patient with PAD will have an expected probability of having significant CAD of:

$$\begin{array}{c} \mathrm{Probability}\;\mathrm{of}\;\mathrm{CAD}=\left\{\left[\left(65\times 0.4\right)+\left(1\times 9\right)+\left(1\times 14\right)\right]\times 2\right\}\;-\;23\\ \mathrm{Probability}\;\mathrm{of}\;\mathrm{CAD}=75\%\left(68.6-81.4\%\right) \end{array}$$

### Curves for the expected probability of CAD

**Figure 4:**
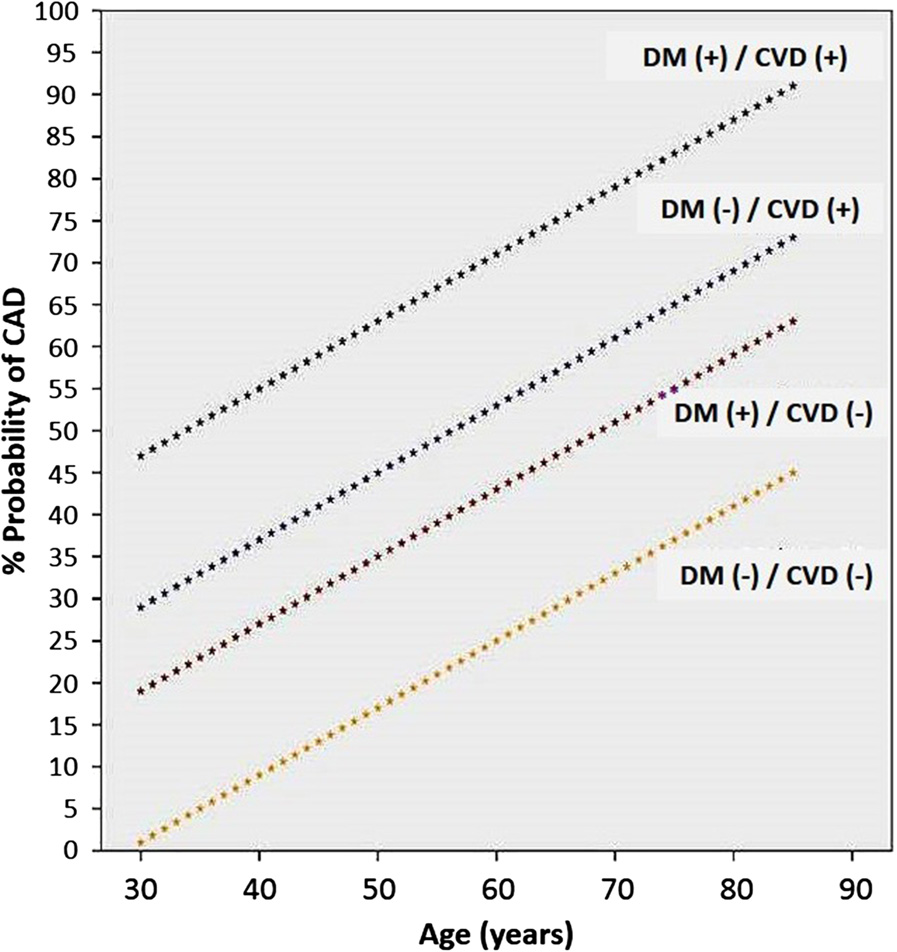
**Expected probability of having significant CAD (y-axis) according to age (x-axis) for patients with (+) or without (−) diabetes (DM) or cardiovascular disease (CVD).** CAD, coronary artery disease; CVD, cardiovascular disease; DM, diabetes mellitus.

Based on the final model, we constructed different curves for the expected probability of significant CAD in renal transplant candidates according to the patient’s age, and the presence or absence of diabetes or CVD. Figure 4 shows those curves.

### Number needed to screen

Based on previous data from our group [2], the estimated 1-year absolute risk of fatal/non-fatal major adverse cardiovascular events (MACE) for patients with CKD stage V is 13% for patients with CAD ≥70% and 2% for patients with CAD <70%. Table 5 shows that, for an estimated 40% prevalence of having significant CAD by the newly developed risk score model, the number needed to screen using coronary angiography to identify one cardiovascular event excess related to CAD is 25.

**Table 5:** **Number needed to screen to identify one cardiovascular event excess per year according to different estimated prevalence of significant CAD**^a^

**Prevalence of CAD ≥70% estimated by the risk score**	**Number of patients with CAD ≥70%**	**Number of patients with CAD <70%**	**Number of events in patients with CAD ≥70% (AR = 13%)**	**Number of events in patients with CAD <70% (AR = 2%)**	**Excess of events in patients with CAD ≥70%**	**Number needed to screen to identify one cardiovascular event**
5%	5	95	0.65	1.90	−1.25	N/A
10%	10	90	1.30	1.80	−0.50	N/A
15%	15	85	1.95	1.70	0.25	400
20%	20	80	2.60	1.60	1.00	100
25%	25	75	3.25	1.50	1.75	57
30%	30	70	3.90	1.40	2.50	40
35%	35	65	4.55	1.30	3.25	31
40%	40	60	5.20	1.20	4.00	25

^a^Based on 100 coronary angiographies. CAD, coronary artery disease; N/A, not applicable; AR, absolute risk.

## Discussion

In this study, we developed and validated a new, simple risk score to predict the probability of significant CAD among asymptomatic, potential renal transplant candidates. This model was defined based on three clinical characteristics previously identified by both the AST and the ERA-EDTA, which indicate intermediate- to high-risk for CAD. Using these three variables, we were able to define a formula to easily predict the pre-test probability of having CAD ≥70%. Moreover, we also constructed curves for the expected probability of CAD for patients with age ranging from 30 to 90 years according to the presence of diabetes or CVD, or both together.

To perform this study, more than 500 renal transplant candidates were assessed not only noninvasively but also by coronary angiography. Owing to ethical reasons, only patients considered ‘at risk’ for having CAD underwent an invasive diagnostic catheterization. Patients at high-risk comprised those with any of the clinical variables (age, diabetes, and CVD), and/or with symptoms or noninvasive testing suggestive of ischemic heart disease. Thus, it came as no surprise that the patients who were considered as high-risk by clinical criteria had such a high prevalence of CAD. For the same reason, all three characteristics entered our model with statistical significance. Despite this selection bias, the final model gave us the relative strength of each variable regarding the risk for CAD. We must emphasize that, in this model, CVD was not only confined (as originally proposed) to the presence of symptomatic CAD, history of a previous myocardial infarction, or signs/symptoms of heart failure. We took a more liberal approach, extending the diagnosis of CVD as a risk factor for CAD if any other extracardiac atherosclerotic disease was present, such as previous stroke or PAD. In previous work, we showed that in high-risk renal transplant candidates, not only were patients with diabetes or previous myocardial infarction at higher risk of significant CAD, but also patients with evidence of extracardiac atherosclerosis, such as PAD [14].

Confirming the assumption that patients included in this study were really at high-risk for CAD, the overall prevalence of coronary stenosis ≥70% was 48.9%. Noteworthy is the fact that we were very strict concerning the diagnosis of CAD in the sense that only significant coronary stenosis was considered in this model. The reason for this approach versus one accepting less severe degrees of stenosis is that we would like to identify those patients with coronary disease more likely to benefit from myocardial revascularization (either percutaneous or surgical) before renal transplantation, following the current guidelines for such an intervention [15]. The identification of less significant CAD, although still related to cardiovascular events, would most likely prompt clinicians to start cardioprotective drugs, such as aspirin, β-blockers, and statins. Thus, our model was devised to seek patients in whom there would be a higher chance for significant CAD that might lead to pre-transplant myocardial revascularization.

The usefulness of a risk score model may be illustrated by the work of Ramanathan *et al*. [5] in which a smaller sample of 97 asymptomatic, diabetic patients with a mean age of 47 years underwent cardiac catheterization prior to renal transplantation. The authors found a 37% overall prevalence of any stenosis ≥70%, reaching 48% in patients with type II diabetes. Following the current guidelines, those patients should be referred for noninvasive testing, and then only if positive, an invasive diagnostic procedure would be recommended. We have previously shown that in 84 renal transplant candidates with diabetes, followed-up for a median of 24 months, cardiac scintigraphy failed to identify those at high-risk for future cardiovascular events [16]. This is in agreement with the study by De Lima *et al*. [2] in which 126 patients with CKD stage V on hemodialysis, followed for a mean of 26 months, the negative predictive value of a transient or fixed defect by SPECT was between 60% and 67% for the diagnosis of CAD; regarding cardiac events, coronary angiography was the best predictor compared with noninvasive testing.

A different approach when screening for significant CAD, specifically in patients with diabetic nephropathy is to perform routine invasive coronary angiography. In a small sample of 40 patients with CKD and type II diabetes, Gang *et al*. [17] studied the value of dobutamine stress echocardiography in detection of CAD. Almost half of the patients had more than 70% lesion in at least one epicardial vessel on coronary angiography; despite its high prevalence, the sensitivity and specificity in identifying CAD was 47% and 95%, respectively, giving an accuracy of this ischemia imaging modality of 72%. The authors concluded that dobutamine stress echocardiography is a poor predictor of CAD in patients with type II diabetes being evaluated for renal transplantation and that coronary angiography should be part of the cardiovascular risk assessment in this population.

Another study by Witczak *et al*. [18], comprising 155 patients with a long history of diabetes and concomitant CKD stages IV or V, found coronary stenosis ≥50% in 69 patients (45%); this resulted in 39 patients being referred for myocardial revascularization (PCI = 17; CABG = 20; PCI + CABG = 2). Based on the high prevalence of significant CAD in this otherwise asymptomatic population and the high rate of myocardial revascularization procedures, the authors concluded and clearly stated that any patient with diabetic nephropathy, regardless of symptoms, should be screened for CAD with coronary angiography before transplantation. This approach was further justified by the demonstration made by the same group [19] that CAD was not a risk factor for mortality in patients with diabetic nephropathy accepted for transplantation when medically treated and revascularized according to standard guidelines. Taken altogether, these data reinforce the paramount importance of a pre-transplant diagnosis of CAD.

Contrary to the latter view, Aalten *et al*. [20] found that in high-risk asymptomatic renal transplant candidates undergoing routine non-invasive cardiac stress testing and coronary angiography only in those with positive results, there was no impact on the incidence of perioperative events and, therefore, they did not recommend such a strategy in all high-risk patients with end-stage renal disease before renal transplantation. As the authors themselves noted, their definition of a ‘high-risk’ patient was quite unique: patients with documented CAD (previous myocardial infarction, PCI, or CABG), for example, were in the same category as those with a body mass index (BMI) greater than 30 kg/m^2^. Moreover, although they did observe a reduction in the number of perioperative cardiac events in those previously screened for CAD compared to a historical, unscreened control group (3.8% versus 7.6%), due to the small number of patients in the trial that difference did not reach statistical significance. Finally, in our study, patients were censored by the time a kidney transplant or a cardiovascular event had occurred, or until the last available visit; this analysis permitted examination of the long-term incidence of events in patients with end-stage renal disease after the diagnosis of significant CAD is made, not restricted to the peritransplant period.

The application of a risk score model as developed in this study may allow clinicians to calculate the expected pre-test probability of having significant CAD on an individual basis and, afterwards, to pursue with no additional investigation at all, to refer patients to noninvasive testing, or to perform an invasive diagnostic procedure. We understand that the best way to address this issue should be by applying noninvasive testing (for instance, cardiac scintigraphy) and to determine its accuracy for the diagnosis of CAD in different pre-test probabilities of CAD. After that and based on the Bayes’ theorem, we would be able to define low-, intermediate-, and high-risk score cut-off points for CAD. At the end, patients at both extremes of the pre-test probability (low/high) will have a very small benefit from noninvasive testing due to a higher rate of false positives in low-risk patients, or due to a higher rate of false negatives in high-risk patients. Moreover, it will be of utmost importance to determine the relationship between the presence of significant CAD and the long-term occurrence of cardiovascular events. Such investigation is presently under way but until these data are provided, at present, it can be left to clinical discretion to perform invasive coronary angiography based on a pre-specified pre-test probability of any given renal transplant candidate to have significant CAD. Based on the number needed to screen to identify one cardiovascular event excess in patients with CAD, we recommend pursuing with coronary angiography if the estimated risk should be at least 40% by the new risk score model. In doing this, for each 25 coronary angiographies performed we would be able to identify one cardiovascular event related to CAD. We should stress that, regardless of the pre-test probability of CAD, if a patient has a clear indication for invasive coronary angiography due to symptoms or results of non-invasive testing suggestive of CAD, then the procedure must be carried on.

## Conclusions

We developed a simple clinical risk score to determine the pre-test probability of any renal transplant candidate of having significant CAD during cardiovascular risk assessment before a patient’s inclusion on waiting lists. We must emphasize, however, that this score must only be applied to those individuals without an unequivocal indication for coronary angiography. This model aims to be a tool in helping those directly involved in the care of patients with end-stage renal disease being considered for transplantation in an attempt to reduce the rate of cardiovascular events that presently hampers the long-term prognosis of such patients.

## Abbreviations

AST: American Society of Transplantation; AUCROC: Area under the receiver operating characteristic curve; BMI: Body mass index; CABG: Coronary artery bypass grafting; CAD: Coronary artery disease; CKD: Chronic kidney disease; CVD: Cardiovascular disease; DAP: Diastolic arterial pressure; DM: Diabetes mellitus; ECG: Electrocardiography; ERA-EDTA: European Renal Association - European Dialysis and Transplant Association; LVEF: Left ventricular ejection fraction; MACE: Major adverse cardiovascular events; MI: Myocardial infarction; PAD: Peripheral artery disease; PCI: Percutaneous coronary intervention; ROC: Receiver operating characteristic; SAP: Systolic arterial pressure; SPECT: Single-photon emission computed tomography; TTE: Transthoracic echocardiography; USRDS: United States Renal Data System.

## Competing interests

The authors declare that they have no competing interests.

## Authors’ contributions

All authors contributed equally to this work. All authors read and approved the final manuscript.
